# University campus breastfeeding, knowledge, and perceptions of support: An exploratory study

**DOI:** 10.1371/journal.pone.0285008

**Published:** 2023-05-26

**Authors:** Allison L. Scott, Ann W. Lambert, Chih-hsuan Wang, Kelly V. Johnson, Jessica Weiss, Tony Stankus

**Affiliations:** 1 Eleanor Mann School of Nursing, University of Arkansas, Fayetteville, Arkansas, United States of America; 2 Auburn University College of Nursing, Auburn University, Auburn, Alabama, United States of America; 3 Auburn University College of Education, Auburn University, Auburn, Alabama, United States of America; 4 St. Luke’s Health System, Kansas City, Missouri, United States of America; 5 University of Arkansas Libraries, University of Arkansas, Fayetteville, Arkansas, United States of America; Johns Hopkins Hospital: Johns Hopkins Medicine, UNITED STATES

## Abstract

Breastfeeding is often considered principally a biological issue but success is impacted by the socio-ecological environment of the lactating parent. Identifying current attitudes towards breastfeeding is essential in the effort toward normalizing breastfeeding in communities, including university campuses. The study explored campus community knowledge, awareness, and attitudes about breastfeeding, including available resources and applicable laws on two university campuses in the southern United States. This cross-sectional, self-reporting study utilized the Iowa Infant Feeding Attitude Scale and an adaptation of the Breastfeeding Behavior Questionnaire to survey a convenience sample. Results revealed decreased awareness of protective laws, availability of private lactation space, and insufficient public appreciation of breastfeeding’s unique advantages to both lactating parent and infant as barriers to breastfeeding. These findings will help develop additional breastfeeding strategies to improve university campus community breastfeeding initiatives.

## Introduction

Breastfeeding is a public health issue important for achieving national and global health goals and improved health outcomes that is supported by the World Health Organization as a key strategy to improve public health [1]. The American Academy of Pediatrics recommends infants be exclusively fed human milk until 6 months old and then continue, adding complementary foods, for 2 years or more [1]. In this study, *breastfeeding* is defined as human milk feeding, whether directly at the breast/chest, previously expressed milk, or a combination thereof [2]. *Exclusive breastfeeding* is defined as human milk feeding with no other liquid or solids [3]. Exclusive breastfeeding for the first 6 months of life provides important nutritional, physical, and psychosocial benefits to both infant and lactating parent [1, 3].

Breastfeeding in public—*public* broadly defined as space outside one’s home—continues to be controversial and contributes to lower breastfeeding rates [4]. Despite public breastfeeding being legal in every state, efforts to encourage acceptance have been necessary [5, 6]. However, public health policies have primarily focused on infant health benefits, with interventions centered around the healthcare system as opposed to home, workplace, or community environments, thus perpetuating stigma [6, 7]. Prior research demonstrates the influence of families, social networks, and community on infant feeding decisions, particularly around breastfeeding in public [5]. Even with health policy support for public breastfeeding and the desire to avoid parental anxiety and feelings of unease, researchers have found experiences of both nursing and expression outside the home to negatively affect breastfeeding rates and duration [5, 7]. Support of public breastfeeding is vital to breastfeeding success and can impact duration [7].

Many lactating parents struggle to continue breastfeeding after entering the workforce and therefore must choose between education/career and the wellness of self and child [8]. Returning to work or attending university courses and maintaining exclusive breastfeeding is a challenge due to barriers such as time, private space, and perception of breastfeeding in public. In prior studies, investigators attributed cessation of breastfeeding to the lactating parent’s return to work [9–12]. University-employed parents reported insufficient break time and inadequate facilities for milk expression storage as barriers [13]. Many higher education campuses are investing in lactation support, but few studies have explored perspectives of the campus community [14].

The federal Break Time for Nursing Mothers law of 2010, covered by the Fair Labor Standards Act, requires all employers with more than 50 employees to provide break time and a private, designated space other than a bathroom for expressing human milk [15, 16]. The 2010 Patient Protection and Affordable Care Act addresses reasonable break times and lactation spaces in the workplace but does not provide universal protection for university students who are not employed full-time by their institution [17]. On public university campuses, students are protected by the Civil Rights Act of 1964, which states that any school or university receiving federal funding cannot discriminate for any reason [18]. Prohibiting a lactating parent to breastfeed or express on campus would be deemed discrimination. Awareness of federal and state breastfeeding laws among university students has not been well studied; one of the few studies found that only 16% of students (undergraduate and graduate combined) at a large southeastern university were aware of laws supporting breastfeeding [18]. Only 3.6% of 139 colleges and universities examined in 2018/2019 had an official policy for lactating students [19].

Universities host faculty, staff, students, and visitors and are thus have unique needs in meeting the challenges of breastfeeding support [19]. One basic need is that of a clean, private space in which to breastfeed. The National Institutes of Health’s general guideline for lactation space recommends a minimum of six lactation expression stations per 1,000 female employees [15]. A study of campus lactation spaces found about half of universities offered at least one dedicated space with an average of 0.33 lactation spaces per 1,000 students [17]. Although research demonstrates benefits, such as cost savings and employee retention, to corporate employers because of workplace lactation support [20], the benefit to universities has not been established [14].

Women have had the legal right to breastfeed in public spaces since 2006 in Alabama [21] and 2007 in Arkansas [22]. Stigma and lack of adequate space remains a major barrier for lactating parents to breastfeed in public. *The PUMP for Nursing Mothers Act*, *2021* passed as an amendment to the omnibus spending package, will expand the requirement for employers to provide certain pumping accommodations for salaried, lactating employees, including pay during milk expression if the employee is also working [23]. The PUMP Act also makes it possible for workers to file a lawsuit seeking monetary compensation if the employer fails to comply. Some states are considering legislation that mandates reasonable accommodations to express human milk or breastfeed on campus, with California being the first state to implement such a law [23].

### Theory

The Socio-Ecological Model (SEM), developed from the early work of Urie Bronfenbrenner [24], guided this study. The Centers for Diseases and Prevention has adapted the SEM for application of health promotion [25]. The SEM can be utilized to determine attitudes, supports, and barriers provided by breastfeeding stakeholders [26, 27]. Health behaviors, such as breastfeeding, are affected by individual factors and the interaction between the individual and actors at interpersonal, community, organization, and policy levels [26]. Barriers to successful breastfeeding include low self-efficacy (individual), lack of peer support (interpersonal), community stigma (community), hospital formula samples (organizational), and lack of specificity in protective laws (policy) [26]. Exploring perspectives of breastfeeding parents pertaining to each level within the SEM is important to determine effective interventions.

This study incorporated each of the five ecological levels. For example, interpersonal factors were incorporated through questions about fathers being left out during breastfeeding. Community factors asked whether breastfeeding is viewed as normal within a participant’s culture, but predominantly focused on their immediate campus environment, given the aims of this study. Organizational and policy factors were explored through questions regarding awareness of current state policies and available lactation resources. The individual level focused on personal attitudes and beliefs. All of these levels must be addressed to enhance the effectiveness of potential future interventions to support breastfeeding in a campus environment.

## Methods

The study had a cross-sectional, self-reporting design. A Qualtrics survey regarding breastfeeding laws and on-campus lactation spaces for faculty, staff, and students at two land-grant universities was utilized to ensure maximum distribution among a large population and enable collaboration between universities. Surveys were used to obtain information related to breastfeeding knowledge, attitudes, and perceptions. The study, *Breastfeeding in Public*: *Knowledge and Perceptions on a University Campus*, received approval by both the University of Arkansas (UAR) Institutional Review Board (IRB), Office of the Provost (approval: 9/8/2020/2007274878) and the Auburn University (AU) IRB, Office of Human Research (approval: 09/29/2021/20-466EX2009). Consent was implied when the participant initiated their response to the survey, as approved by both university IRB boards. Participants could opt out of the survey at any time.

### Setting

Participants comprised students and employees (faculty and staff) from UAR located in Fayetteville, Arkansas and AU located in Auburn, Alabama. UAR has approximately 23,000 students and 2,400 faculty and staff members [28]. While the 2022 CDC *Breastfeeding Report Card* reports that nationally 83.2% of infants in 2019 were ever breastfed, only 55.8% were still breastfeeding at 6 months old and 36.9% at 12 months old [29]. In Arkansas, only 74.9% were ever breastfed; 46.5% were still breastfeeding at 6 months old.

AU has an enrollment of approximately 30,000 students and 6,400 faculty and staff members [30]. Only 71.1% of infants born in Alabama in 2019 were ever breastfed; 37.7% were still breastfeeding 6 months old [29].

Both universities subscribe to federal employment policies providing faculty and staff appropriate time, at least 13 private lactation spaces each, and available refrigeration to support breastfeeding practices. Lactating parents enrolled as students at both universities have access to campus lactation spaces. Supported by their respective undergraduate nursing departments, both UAR and AU provide designated lactation support space at several athletic campus venues. An online map of each campus provides locations of permanent lactation spaces.

### Sample

The target population was a convenience sample of all undergraduate and graduate students, faculty, and staff members at UAR and AU. Inclusion criteria were age 18 years or older, a current student, faculty, or staff member (any employee) at either university, and consent for participation in the study. The study invitation stated participation was voluntary and completion of the 10-minute survey through Qualtrics implied consent. No exclusion criteria applied. As an incentive to complete the survey, participants had the option to provide their email for the chance to receive one of 10 $50 Amazon gift cards. Emails were de-identified from the survey after the drawing.

**Fig 1:**
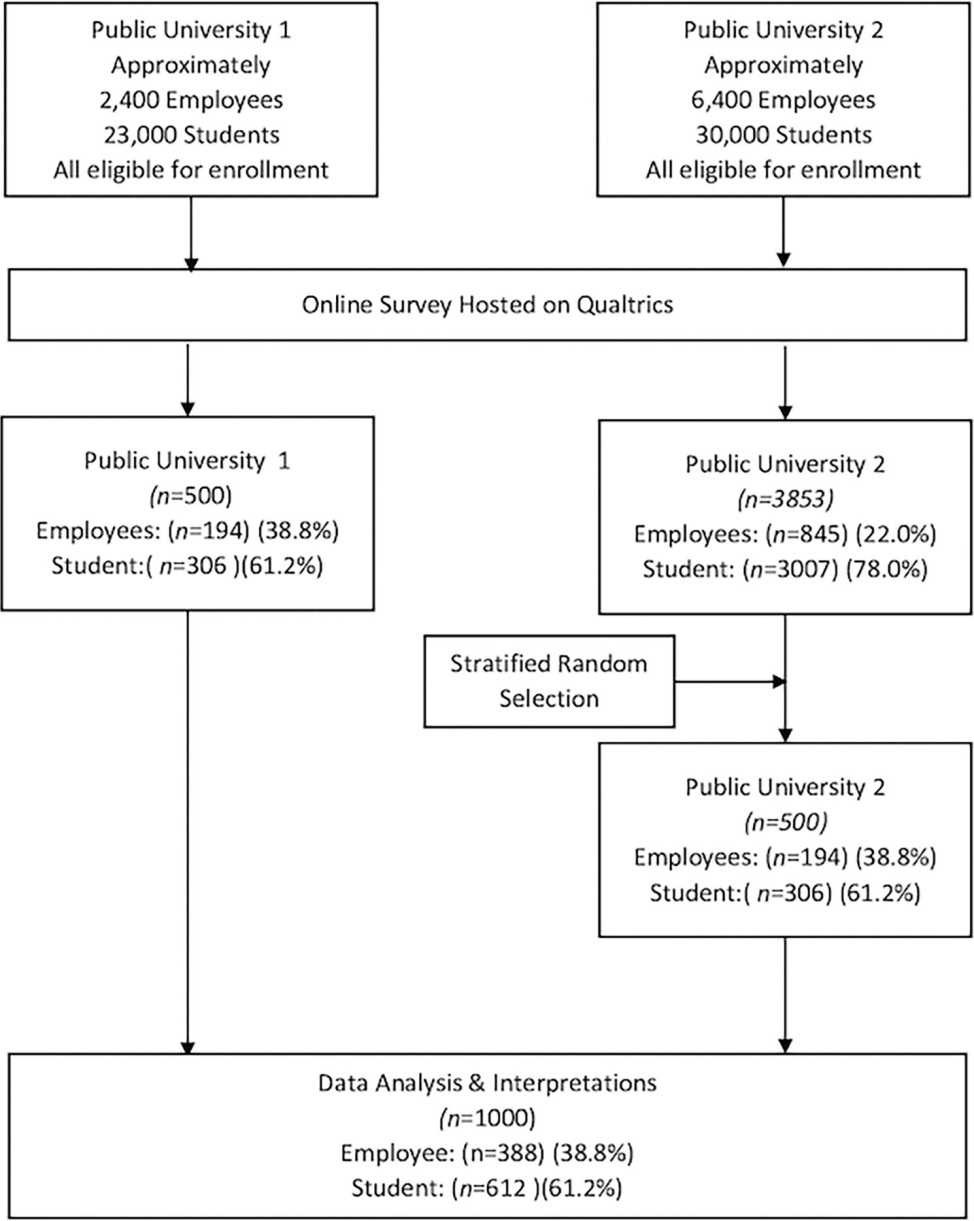
Campus breastfeeding sample selection flow diagram.

There were 623 returned responses at UAR. Of these, 500 responses were received, with a survey usable (all questions completed) rate of 80.3%. There were 4,028 returned responses at AU with 3,853 fully completed surveys, yielding a survey completion rate of 95.7%. Due to large differences in the quantity of returned responses, we utilized stratified random sampling procedure to select 500 responses from AU participants, randomly but with the same proportion of students, faculty, and staff as comprised the UAR responses. Using power analysis with a significance level of .05, power at .80, and medium effect size Cohen’s *d* at 0.5, at least 51 participants per research site with 102 total participants was sufficient for the study. The sample size of 1000 participants exceeded adequacy for this study. Fig 1 reflects the flow diagram for this study.

### Measurement

Participants were asked demographic questions including status (undergraduate or graduate student, faculty, or staff), gender (female, male, gender diverse, or decline to answer), ethnicity, age, and if they had children. The second part of the survey utilized, with author permission, the Iowa Infant Feeding Attitudes Scale (IIFAS), a validated tool to measure maternal attitudes toward infant feeding [31]. The IIFAS is a 17 question, 5-point Likert scale tool that can be easily administered to measure breastfeeding attitudes and is a strong predictor of breastfeeding behavior, including duration. A higher score on the IIFAS indicates a more positive attitude toward breastfeeding [31]. Additional questions were adapted from the Breastfeeding Behavior Questionnaire (BBQ) with five items based on the original situational question but modified in a non-scenario form using a 5-point Likert scale. The BBQ, developed by Libbus [32], examines attitudes and beliefs influencing infant-feeding choice among populations with varied demographics, with a lower score indicating an increased positive attitude and more accurate knowledge concerning breastfeeding. A lower score in the adapted BBQ also indicated a more positive attitude.

The Qualtrics survey consisted of five questions about attitudes toward breastfeeding in public, 16 questions about perceptions of the benefits of breastfeeding, five knowledge questions about campus lactation spaces, and five questions regarding state and federal laws pertaining to breastfeeding. Questions regarding lactation spaces, laws, and personal attitude were generated by the primary investigator, based on existing state laws and university campus resources in place at the time of the study. Two questions about personal attitudes toward breastfeeding, unrelated to public breastfeeding, and two knowledge questions about potential benefits of breastfeeding were also included.

Both attitude and perception subscales were 5-point Likert-type scales. Participants responded to each item from 1 = *Strongly Disagree* to 5 = *Strongly Agree*. To validate the psychometric property of the measures, reliability analysis to calculate internal consistency (Cronbach’s Alpha) was conducted. Cronbach’s Alpha provides a value between 0 to 1 to indicate the relationship among items within a measure [33]. A value of .70 or above is considered good reliability and indicates the items within the same measure are measuring the same construct [34]. Results from reliability analysis indicated that Cronbach’s Alpha was .80 for the attitude subscale and .82 for the perception subscale, indicating good reliability.

The knowledge subscale contained questions where participants responded to each item by choosing *True*, *False*, or *Don’t Know*. The correct answers were coded as 1, while incorrect answers or *Don’t Know* was coded as 0. The Cronbach’s Alpha for knowledge about campus resources was .74 and .72 for knowledge about state and federal laws, indicating acceptable reliability. Nursing professionals at both sites reviewed the surveys to secure face and content validity. The complete survey is provided in Appendices 1 and 2.

### Data collection

From October 19, 2020, to March 1, 2021, students, faculty and staff at both campuses were recruited through email and social media generated by the UAR College of Education and Health Professions and AU Office of Institutional Research. The email included a brief introduction to the study and the Qualtrics survey link. Participants had an opportunity to review study information and consent before beginning the survey. A reminder email was distributed one week later. Due to the large study population, only one reminder was distributed. Social media solicitations were posted several times at both study sites.

### Data analysis

Total scores of the attitude, perception, and knowledge subscales were calculated for each participant and then analyzed using independent sample *t*-tests to compare differences in responses between participants from both universities as well as several subgroups. The personal attitude and knowledge of benefits questions were multiple-choice questions. Chi-square Independence Tests were used to compare the differences between each option in these questions within each university, between participants from both universities, and between employees and students. When Cohen’s *d* was used to calculate effect size, with effect sizes classified as small (*d* = 0.2), medium (*d* = 0.5), and large (*d* ≥ 0.8); effect size reported with Cramer’s *V* is classified as weak (*V* ≤ 0.2), moderate (0.2 < *V* ≤ 0.6), and strong (*V* > 0.6).

## Results

### Demographic

The average age of participants was 28.4 years old (*SD* = 11.04). Table 1 provides a full demographic breakdown of participants. Notably, at UAR, 85.4% of participants were White (*n* = 427), compared to only 72% on campus as a whole [28]. Female (90.2%) participation far outweighed male (9.0%). Just over one-third (37.0%) of participants had children. At AU, 85.0% of participants were White (*n* = 424), closer to the overall campus demographics of 78% [30]. Males were better represented, accounting for almost one-third (29.8%) of responses. Fewer participants reported having children (28.6%) compared with UAR {Table 1}.

**Table 1:** Demographics of participants by site.

	UAR			AU			Overall (*n* = 1000)
	Employee (*n* = 194)	Student (*n* = 306)	Total (*n* = 500)	Employee (*n* = 194)	Student (*n* = 306)	Total (*n* = 500)	
	*n* (%)	*n* (%)	*n* (%)	*n* (%)	*n* (%)	*n (%)*	*n (*%)
Ethnicity							
White	169 (87.1)	258 (84.3)	427 (85.4)	164 (84.5)	260 (85.0)	424 (85.0)	851 (85.2)
Hispanic	5 (2.6)	12 (3.9)	17 (3.4)	7 (3.6)	10 (3.3)	17 (3.4)	34 (3.4)
African American	7 (3.6)	7 (2.3)	14 (2.8)	7 (3.6)	14 (4.6)	21 (4.2)	35 (3.5)
Native American	6 (3.1)	1 (0.3)	7 (1.4)	1 (0.5)	0 (0.0)	1 (0.2)	8 (0.8)
Asian/Pacific	4 (2.1)	12 (3.9)	16 (3.2)	9 (4.6)	18 (5.9)	27 (5.4)	43 (4.3)
Other	3 (1.5)	16 (5.2)	19 (3.8)	5 (2.6)	4 (1.3)	9 (1.8)	28 (2.8)
Gender							
Male	23 (11.9)	22 (7.2)	45 (9.0)	47 (24.2)	102 (33.3)	149 (29.8)	194 (19.4)
Female	170 (87.6)	281 (91.8)	451 (90.2)	141 (72.7)	197 (64.4)	338 (67.6)	789 (78.9)
Gender Diverse	1 (0.5)	1 (0.3)	2 (0.4)	1 (0.5)	2 (0.7)	3 (0.6)	5 (0.5)
Prefer not to answer	0 (0.0)	2 (0.7)	2 (0.4)	5 (2.6)	5 (1.6)	10 (2.0)	12 (1.2)
Children							
Yes	152 (78.4)	33 (10.8)	185 (37.0)	128 (66.0)	15 (4.9)	143 (28.6)	328 (32.8)
No	42 (21.6)	273 (89.2)	315 (63.0)	66 (34.0)	291 (95.1)	357 (71.4)	672 (67.2)

### Comparison of UAR and AU

Students and employees at UAR demonstrated somewhat more positive attitudes and perceptions toward breastfeeding in public (*M* = 3.97, *SD* = 0.77; *M* = 3.62, *SD* = 0.50, respectively) compared with AU students (*M* = 3.74, *SD* = 0.88; *t*(998) = 4.40, *p* < .001, Cohen’s *d* = 0.28) and employees (*M* = 3.49, *SD* = 0.46; *t*(998) = 4.44, *p* < .001, Cohen’s *d* = 0.28).

UAR participants were more knowledgeable about their campus breastfeeding resources (*M* = 1.84, *SD* = 1.59) and state and federal laws (*M* = 2.55, *SD* = 1.66) compared with AU participants’ knowledge of campus resources (*M* = 1.33, *SD* = 1.44; *t* (998) = 5.40, *p* < .001, Cohen’s *d* = 0.34) and laws (*M* = 1.98, *SD* = 1.63; *t*(998) = 5.50, *p* < .001, Cohen’s *d* = 0.35).

Participants at UAR (*n* = 469, 93.8%) demonstrated similar attitudes about an ethical right to breastfeed in public as those at AU (*n* = 449, 89.8%). However, moderately more participants at UAR (*n* = 439, 87.7%) indicated breastfeeding laws mattered to them compared to AU participants (*n* = 377, 75.4%) (χ^2^(2) = 28.20, *p* < .001, Cramer’s *V* = 0.17).

When comparing UAR and AU data on each item in the knowledge scale, differences in responses to most campus, state law, and federal law items were statistically significant, except for questions regarding breastfeeding space provided at university athletic events. In general, participants at UAR were more likely to answer these questions correctly, with small or large effect size. Participants’ responses on the knowledge and personal attitude scales are displayed by study site {Table 2}.

**Table 2:** Knowledge and personal attitude scales by site.

	UAR (*n* = 500)			AU (*n* = 500)			χ^2^ (2)	*p*	Cramer’s *V*
	*True n (%)	False n (%)	DK n (%)	*True n (%)	False n (%)	DK n (%)			
Campus									
Designated breastfeeding areas exist across campus.	232 (46.4)	27 (5.4)	241 (48.2)	153 (30.6)	30 (6.0)	317 (63.4)	26.72	< .001	.16
There are at least 13 designated breastfeeding areas on campus.	69 (13.8)	32 (6.4)	399 (79.8)	38 (7.6)	22 (4.4)	440 (88.0)	12.74	.002	.11
Based on the State law, designated space, including employee office space, must be available on campus.	287 (57.4)	11 (2.2)	202 (40.4)	224 (44.8)	16 (3.2)	260 (52.0)	15.97	< .001	.13
A campus map of breastfeeding areas is available online.	124 (24.8)	34 (6.8)	342 (68.4)	69 (13.8)	20 (4.0)	411 (82.2)	25.63	< .001	.16
Private breastfeeding space at some university athletic events is currently offered.	211 (42.1)	16 (3.2)	274 (54.7)	179 (35.8)	15 (3.0)	306 (61.2)	4.38	.11	.07
State Law									
Your employer is legally required to give you break time to breastfeed, but this break time may not be paid in all workplaces.	265 (53.0)	14 (2.8)	221 (44.2)	184 (36.8)	16 (3.2)	300 (60.0)	26.73	< .001	.16
Your employer is legally required to make a reasonable effort to provide a clean, secure, and private area to breastfeed apart from the bathroom stall.	281 (56.2)	22 (4.4)	197 (39.4)	214 (42.8)	31 (6.2)	255 (51.0)	18.04	< .001	.13
A woman has the legal right to breastfeed in public.	336 (67.2)	11 (2.2)	153 (30.6)	292 (58.3)	14 (2.8)	195 (38.9)	8.33	.02	.09
Before taking this survey, I was aware there are laws related to breastfeeding in the State of Arkansas.	235 (46.9)	198 (39.5)	68 (13.6)	178 (35.6)	226 (45.2)	96 (19.2)	14.38	.001	.12
Federal Law									
The federal law “Break time for nursing mother’s provision” states break time to express breast milk be provided to an employee, up to 1 year after the child’s birth.	160 (31.9)	2 (0.4)	339 (67.7)	122 (24.4)	3 (0.6)	375 (75.0)	6.99	.03	.08
Personal Attitude									
A woman has the ethical right to breastfeed in public.	469 (93.8)	12 (2.4)	19 (3.8)	449 (89.8)	19 (3.8)	32 (6.4)	5.33	.07	.07
Breastfeeding laws matter to me.	439 (87.7)	32 (6.3)	30 (6.0)	377 (75.4)	45 (9.0)	78 (15.6)	28.20	< .001	.17

Note

* indicates correct answer

When asked about the most beneficial choice to feed an infant less than 6 months, almost half of the participants at UAR (*n* = 226, 45.2%) correctly indicated that exclusive or only breastfeeding is most beneficial, significantly more than AU participants (*n* = 145, 29.0%; χ^2^(4) = 47.94, *p* < .001). Almost one-third of AU participants indicated they did not know the answer (*n* = 154, 30.8%). However, the same number of participants at UAR and AU (*n* = 188, 37.6%) incorrectly identified predominant or mostly breastfeeding as the most beneficial choice. Compared with AU (*n* = 153, 39.6%), significantly more participants at UAR (*n* = 229, 45.8%) correctly identified lower pediatric health costs as an economic benefit (χ^2^(2) = 25.99, *p* < .001, Cramer’s *V* = .16) {Table 3}.

**Table 3:** Benefit choice and economic benefits by site.

	UAR (*n* = 500)	AU (*n* = 500)	χ^2^ (2)	*p*	Cramer’s *V*
	*n* (%)	*n* (%)			
When feeding an infant less than 6 months, the most beneficial choice is:			47.94	< .001	.22
Partial breastfeeding	13 (2.6)	11 (2.2)			
Predominant or mostly breastfeeding	188 (37.6)	188 (37.6)			
*Exclusive or only breastfeeding	226 (45.2)	145 (29.0)			
No breastfeeding	1 (0.2)	2 (0.4)			
I don’t know	72 (14.4)	154 (30.8)			
Which of the following is true regarding breastfeeding and economic benefits?			25.99	< .001	.16
*It has been estimated that the U.S. economy could save over $10 billion per year in pediatric health cost, if 90% of women breastfed for the first year of life.	228 (45.8)	153 (30.6)			
There is little evidence that healthcare costs would change if the majority of mothers breastfed in the U.S.	30 (6.0)	28 (5.6)			
I don’t know.	240 (48.2)	319 (63.8)			

Note

* indicates correct answer.

### Comparison of UAR and AU females

Due to a significant difference in the number of male participants between UAR and AU, analysis of female participants was conducted to eliminate potential bias. Compared with female AU participants, female UAR participants demonstrated more positive attitudes and perceptions toward breastfeeding in public (UAR; *n* = 422, 93.6%, UA; *n* = 306, 90.5%) and were also more knowledgeable about breastfeeding resources on campus and state and federal laws (UAR; *n* = 201, 46.6%; AU (*n* = 96, 28.4%).

When asking if a woman has the ethical right to breastfeed in public, both UAR and AU females responded similarly, with 93.6% (*n* = 422) of female UAR participants and 90.5% (*n* = 306) of female AU participants responding *True*. When asked if “Breastfeeding laws matter to me,” more female UAR participants agreed (*n* = 399, 88.5%) compared to female AU participants (*n* = 275, 81.4%). Approximately half of all participants at UAR (*n* = 219, 48.3%) and just over one-third of all participants at AU (n = 131, 38.8%) were aware of existing state laws regarding breastfeeding. More than half of UAR participants (*n* = 247, 54.8%) and less than half of AU participants (*n* = 145, 42.9%) responded that an employer is legally required to allow break time for pumping in the workplace. Female UAR and AU participants’ responses differed with statistical significance to each item in the knowledge scale, except when asked if they were aware of private breastfeeding space offered during university athletic events. Overall, UAR participants were more likely to correctly answer these items in the knowledge scale than AU participants{Tables 4 & 5}.

**Table 4:** Knowledge scale of female participants.

	UAR (*n* = 451)			AU (*n* = 338)			χ^2^ (2)	*p*	Cramer’s *V*
	*True *n* (%)	False *n* (%)	DK *n* (%)	*True *n* (%)	False *n* (%)	DK *n* (%)			
Campus									
Designated breastfeeding areas exist across campus.	210 (46.6)	22 (4.9)	219 (48.6)	96 (28.4)	25 (7.4)	217 (64.2)	27.04	< .001	.19
There are at least 13 designated breastfeeding areas on campus.	66 (14.6)	26 (5.8)	359 (79.6)	18 (5.3)	19 (5.6)	300 (88.8)	17.68	< .001	.15
Based on the State law, designated space, including employee office space, must be available on campus.	264 (58.5)	11 (2.4)	176 (39.0)	163 (48.2)	12 (3.6)	163 (48.2)	8.42	.02	.10
A campus map of breastfeeding areas is available online.	114 (25.3)	28 (6.2)	309 (68.5)	40 (11.8)	16 (4.7)	282 (83.4)	24.38	< .001	.18
Private breastfeeding space at some university athletic events is currently offered.	191 (42.4)	14 (3.1)	245 (54.3)	129 (38.2)	11 (3.3)	198 (58.6)	1.47	.48	.04
State Law									
Your employer is legally required to give you break time to breastfeed, but this break time may not be paid in all workplaces.	247 (54.8)	13 (2.9)	191 (42.4)	145 (42.9)	12 (3.6)	181 (53.6)	10.89	.004	.12
Your employer is legally required to make a reasonable effort to provide a clean, secure, and private area to breastfeed apart from the bathroom stall.	260 (57.6)	21 (4.7)	170 (37.7)	161 (47.6)	22 (6.5)	155 (45.9)	7.98	.02	.10
A woman has the legal right to breastfeed in public.	315 (69.8)	9 (2.0)	127 (28.2)	204 (60.4)	6 (1.8)	127 (37.6)	8.02	.02	.10
Before taking this survey, I was aware there are laws related to breastfeeding in the State of AR/AL.	218 (48.3)	170 (37.7)	62 (13.7)	131 (38.8)	157 (46.4)	50 (14.8)	7.73	.02	.10
Federal Law									
The federal law “Break time for nursing mother’s provision” states break time to express breast milk be provided to an employee, up to 1 year after the child’s birth.	148 (32.8)	2 (0.4)	301 (66.7)	89 (26.3)	2 (0.6)	247 (73.1)	3.91	.14	.07
Personal Attitude									
A woman has the ethical right to breastfeed in public.	422 (93.6)	9 (2.0)	19 (4.2)	306 (90.5)	14 (4.1)	17 (5.0)	3.53	.17	.07
Breastfeeding laws matter to me.	399 (88.5)	24 (5.3)	25 (5.5)	275 (81.4)	21 (6.2)	42 (12.4)	12.17	.002	.12

Note

* indicates correct answer

**Table 5:** Benefit choice and economic benefits by female participants.

	UAR (*n* = 451)	AU (*n* = 338)	χ^2^	*p*	Cramer’s *V*
	*n* (%)	*n* (%)			
When feeding an infant less than 6 months, the most beneficial choice is:			*df* = 4 27.25	< .001	.19
Partial breastfeeding	13 (2.9)	9 (2.7)			
Predominant or mostly breastfeeding	169 (37.5)	131 (38.8)			
*Exclusive or only breastfeeding	210 (46.6)	111 (32.8)			
No breastfeeding	1 (0.2)	0 (0.0)			
I don’t know	58 (12.9)	87 (25.7)			
Which of the following is true regarding breastfeeding and economic benefits?			*df* = 2 14.29	< .001	.14
*It has been estimated that the U.S. economy could save over $10 billion per year in pediatric health cost, if 90% of women breastfed for the first year of life.	204 (45.2)	110 (32.5)			
There is little evidence that healthcare costs would change if the majority of mothers breastfed in the U.S.	30 (6.7)	21 (6.2)			
I don’t know.	216 (47.9)	207 (61.2)			

Note

* indicates correct answer

Comparing responses given by female participants did not differ statistically from the overall analysis between UAR and AU. Therefore, although there were more male participants at AU, this factor did not influence the results and interpretations.

### Comparison of students and employees

Prior research found student awareness of breastfeeding laws was lower than faculty and staff [18]. To explore this finding, a comparison of students and employees was conducted. Participating employees (*M* = 4.00, *SD* = 0.83) demonstrated more positive attitudes and perceptions toward breastfeeding in public than students (*M* = 3.71, *SD* = 0.51). Employees were also more knowledgeable than students about breastfeeding resources on campus along with state and federal laws (*M* = 2.03, *SD* = 1.58; *M* = 2.64, *SD* = 1.76, respectively).

When asking if a woman has the ethical right to breastfeed in public, both employees and students responded similarly, with 92.3% (*n* = 358) of employees and 91.5% (*n* = 560) of students responding *True*. When asked if “Breastfeeding laws matter to me,” their responses were similar, with 84.8% (*n* = 329) of employees and 79.4% (*n* = 486) of students agreeing breastfeeding laws matter. Only about half of participants were aware of existing state laws regarding breastfeeding and that an employer is legally required to allow break time for pumping in the workplace (UAR; *n* = 191, 49.2% and AU; *n* = 258, 42.2%). When comparing student and employee responses to each item in the knowledge scale, responses to most of these items differed with statistical significance, except items asking if they were aware their employer is legally required to give breastfeeding parents break time to breastfeed and the legal right to breastfeed in public. Overall, employee participants were more likely to correctly answer these items in the knowledge scale than student participants {Table 6}.

**Table 6:** Knowledge scale by role.

	Employee (*n* = 388)			Student (*n* = 612)			χ^2^ (2)	*p*	Cramer’s *V*
	*True *n* (%)	False *n* (%)	DK *n* (%)	*True *n* (%)	False *n* (%)	DK *n* (%)			
Campus (Cronbach’s alpha = .74)									
Designated breastfeeding areas exist across campus.	209 (53.9)	13 (3.4)	166 (42.8)	176 (28.8)	44 (7.2)	392 (64.1)	64.27	< .001	.25
There are at least 13 designated breastfeeding areas on campus.	55 (14.2)	11 (2.8)	322 (82.9)	52 (8.5)	43 (7.0)	517 (84.5)	14.97	.001	.12
Based on the State law, designated space, including employee office space, must be available on campus.	240 (61.9)	13 (3.4)	135 (34.8)	271 (44.3)	14 (2.3)	327 (53.4)	33.10	< .001	.18
A campus map of breastfeeding areas is available online.	103 (26.5)	9 (2.3)	276 (71.1)	90 (14.7)	45 (7.4)	477 (77.9)	29.85	< .001	.17
Private breastfeeding space at some university athletic events is currently offered.	180 (46.4)	6 (1.5)	202 (52.1)	209 (34.2)	25 (4.1)	378 (61.7)	17.81	< .001	.13
State Law (Cronbach’s alpha = .72)									
Your employer is legally required to give you break time to breastfeed, but this break time may not be paid in all workplaces.	191 (49.2)	11 (2.8)	186 (47.9)	258 (42.2)	19 (3.1)	335 (54.7)	4.81	.09	.07
Your employer is legally required to make a reasonable effort to provide a clean, secure, and private area to breastfeed apart from the bathroom stall.	190 (48.9)	8 (2.1)	138 (35.6)	253 (41.3)	45 (7.4)	314 (51.3)	46.78	< .001	.22
A woman has the legal right to breastfeed in public.	256 (65.9)	7 (1.8)	125 (32.3)	372 (60.8)	18 (2.9)	222 (36.3)	3.28	.19	.06
Before taking this survey, I was aware there are laws related to breastfeeding in the State of AR/AL.	213 (54.9)	109 (28.1)	66 (17.0)	200 (32.6)	315 (51.4)	98 (16.0)	59.24	< .001	.24
Federal Law									
The federal law “Break time for nursing mother’s provision” states break time to express breast milk be provided to an employee, up to 1 year after the child’s birth.	123 (31.8)	0 (0.0)	265 (68.2)	158 (25.8)	5 (0.8)	449 (73.4)	7.04	.03	.08
Personal Attitude									
A woman has the ethical right to breastfeed in public.	358 (92.3)	16 (4.1)	14 (3.6)	355 (91.5)	10 (2.5)	24 (6.1)	4.93	.09	.07
Breastfeeding laws matter to me.	329 (84.8)	28 (7.2)	31 (8.0)	308 (79.4)	31 (7.9)	49 (12.7)	5.67	.06	.08

Note

* indicates correct answer

When asked about the beneficial choice of feeding an infant less than 6 months, student and employee responses differed significantly (χ^2^(4) = 47.65, *p* < .001, Cramer’s *V* = .22). Although a similar percentage of participating employees (*n* = 113, 34.3%) and students (*n* = 243, 39.7%) incorrectly identified predominant or mostly breastfeeding as the most beneficial choice for infants younger than 6 months, significantly more employees (*n* = 194, 49.9%) than students (*n* = 181, 29.6%) indicated that exclusive breastfeeding was the most beneficial choice, reinforcing the finding that more student participants (*n* = 164, 26.8%) did not know the answer compared with employee participants (*n* = 62, 16.0%). Regarding economic benefits, more employee participants (*n* = 174, 44.8%) correctly indicated economic benefits of breastfeeding in relation to pediatric health costs than student participants (*n* = 208, 34.0%) (χ^2^(2) = 11.77, *p* = .003, Cramer’s *V* = .11). The majority of student participants (*n* = 366, 59.8%) indicated they did not know the answer to this question {Table 7}.

**Table 7:** Benefit choice and economic benefits by role.

	Employee (*n* = 388)	Student (*n* = 612)	χ^2^	*p*	Cramer’s *V*
	*n* (%)	*n* (%)			
When feeding an infant less than 6 months, the most beneficial choice is:			*df* = 4 47.65	< .001	.22
Partial breastfeeding	2 (0.5)	22 (3.6)			
Predominant or mostly breastfeeding	133 (34.3)	243 (39.7)			
*Exclusive or only breastfeeding	190 (49.9)	181 (29.6)			
No breastfeeding	1 (0.3)	2 (0.3)			
I don’t know	62 (16.0)	164 (26.8)			
Which of the following is true regarding breastfeeding and economic benefits?			*df* = 2 11.77	.003	.11
*It has been estimated that the U.S. economy could save over $10 billion per year in pediatric health cost, if 90% of women breastfed for the first year of life.	173 (44.8)	208 (34.0)			
There is little evidence that healthcare costs would change if the majority of mothers breastfed in the U.S.	20 (5.2)	38 (6.2)			
I don’t know.	193 (50.0)	366 (59.8)			

Note

* indicates correct answer

### Comparison of UAR and AU employees

There was no statistical difference between employees at each institution regarding positive attitudes and perceptions toward breastfeeding in public and knowledge about breastfeeding resources on campus and state/federal laws. Overall differences between UAR and AU remain when employees between the sites are compared, with the exception of UAR being more aware of campus designated breastfeeding areas and the correlating campus map than AU employees (32, 16.5%), with both reporting low awareness. As previously stated, this did not vary from overall results, comparing the two sites {Tables 8 & 9}.

**Table 8:** Chi-square tests for knowledge scale between UAR and AU employees.

	UAR (*n* = 194)			AU (*n* = 194)			χ^2^ (2)	*p*	Cramer’s *V*
	*True *n* (%)	False *n* (%)	DK *n* (%)	*True *n* (%)	False *n* (%)	DK *n* (%)			
Campus									
Designated breastfeeding areas exist across campus.	123 (63.4)	2 (1.0)	69 (35.6)	86 (44.3)	11 (5.7)	97 (50.0)	17.50	< .001	.21
There are at least 13 designated breastfeeding areas on campus.	32 (16.5)	5 (2.6)	157 (80.9)	23 (11.9)	6 (3.1)	164 (84.5)	1.71	.42	.07
Based on the State law, designated space, including employee office space, must be available on campus.	128 (66.0)	5 (2.6)	61 (31.4)	112 (57.7)	8 (4.1)	74 (38.1)	3.01	.22	.09
A campus map of breastfeeding areas is available online.	71 (36.6)	4 (2.1)	119 (61.3)	32 (16.5)	5 (2.6)	157 (80.9)	20.11	< .001	.23
Private breastfeeding space at some university athletic events is currently offered.	93 (47.9)	3 (1.5)	98 (50.5)	87 (44.8)	3 (1.5)	104 (53.6)	0.38	.83	.03
State Law									
Your employer is legally required to give you break time to breastfeed, but this break time may not be paid in all workplaces.	106 (54.6)	4 (2.1)	84 (43.3)	85 (43.8)	7 (3.6)	102 (52.6)	4.87	.09	.11
Your employer is legally required to make a reasonable effort to provide a clean, secure, and private area to breastfeed apart from the bathroom stall.	130 (67.0)	3 (1.5)	61 (31.4)	112 (57.7)	5 (2.6)	77 (39.7)	3.69	.16	.10
A woman has the legal right to breastfeed in public.	135 (69.6)	4 (2.1)	55 (28.4)	120 (61.9)	3 (1.5)	70 (36.1)	2.82	.24	.09
Before taking this survey, I was aware there are laws related to breastfeeding in the State of AR/AL.	119 (61.3)	43 (22.2)	32 (16.5)	94 (48.5)	66 (34.0)	34 (17.5)	7.85	.02	.14
Federal Law									
The federal law “Break time for nursing mother’s provision” states break time to express breast milk be provided to an employee, up to 1 year after the child’s birth.	68 (35.1)	0 (0.0)	125 (64.4)	55 (28.4)	0 (0.0)	139 (71.6)	2.11	.15	.07
Personal Attitude									
A woman has the ethical right to breastfeed in public.	184 (94.8)	5 (2.6)	5 (2.6)	174 (89.7)	11 (5.7)	9 (4.6)	3.67	.16	.10
Breastfeeding laws matter to me.	176 (90.7)	8 (4.1)	10 (5.2)	152 (78.4)	20 (10.3)	21 (10.8)	10.80	.01	.17

Note

* indicates correct answer

**Table 9:** Chi-square test results for benefit choice and economic benefits between UAR and AU employees.

	UAR (*n* = 194)	AU (*n* = 194)	χ^2^	*p*	Cramer’s *V*
	*n* (%)	*n* (%)			
When feeding an infant less than 6 months, the most beneficial choice is:			*df* = 4 27.21	< .001	.27
Partial breastfeeding	0 (0.0)	2 (1.0)			
Predominant or mostly breastfeeding	58 (29.9)	75 (38.7)			
*Exclusive or only breastfeeding	118 (60.8)	72 (37.1)			
No breastfeeding	0 (0.0)	1 (0.5)			
I don’t know	18 (9.3)	44 (22.7)			
Which of the following is true regarding breastfeeding and economic benefits?			*df* = 2 12.06	.002	.18
*It has been estimated that the U.S. economy could save over $10 billion per year in pediatric health cost, if 90% of women breastfed for the first year of life.	103 (53.1)	70 (36.1)			
There is little evidence that healthcare costs would change if the majority of mothers breastfed in the U.S.	8 (4.1)	12 (6.2)			
I don’t know.	81 (41.8)	112 (57.7)			

Note

* indicates correct answer

### Comparison of UAR and AU students

Participating UAR students demonstrated more positive attitudes and perceptions toward breastfeeding in compared with AU students. No statistical difference existed comparing campus resources, except increased awareness of designated campus lactation space and their associated laws by UAR students. UAR students were also more knowledgeable about state and federal laws than AU students.

When comparing the UAR and AU students’ responses of each item in the Knowledge scale, UAR students responded to most of these items differed with statistical significance, except the items asking if private breastfeeding space offered in university athletic events. Overall, UAR participating students were more likely to correctly answer these items in the Knowledge scale than AU student participants, including ideal beneficial feeding choice and economic healthcare benefits {Tables 10 & 11}. The results did not differ from the comparison of UAR and AU female only participants {Tables 10 & 11}.

**Table 10:** Chi-square tests for knowledge scale between UAR and AU student.

	UAR (*n* = 306)			AU (*n* = 306)			χ^2^ (2)	*p*	Cramer’s *V*
	*True *n* (%)	False *n* (%)	DK *n* (%)	*True *n* (%)	False *n* (%)	DK *n* (%)			
Campus									
Designated breastfeeding areas exist across campus.	109 (35.6)	25 (8.2)	172 (56.2)	67 (21.9)	19 (6.2)	220 (71.9)	16.72	< .001	.17
There are at least 13 designated breastfeeding areas on campus.	37 (12.1)	27 (8.8)	242 (79.1)	15 (4.9)	16 (5.2)	275 (89.9)	14.23	< .001	.15
Based on the State law, designated space, including employee office space, must be available on campus.	159 (52.0)	6 (2.0)	141 (46.1)	112 (36.6)	8 (2.6)	186 (60.8)	14.63	< .001	.16
A campus map of breastfeeding areas is available online.	53 (17.3)	30 (9.8)	223 (72.9)	37 (12.1)	15 (4.9)	254 (83.0)	9.86	.01	.13
Private breastfeeding space at some university athletic events is currently offered.	117 (38.2)	13 (4.2)	175 (57.2)	92 (30.1)	12 (3.9)	202 (66.0)	4.96	.08	.09
State Law									
Your employer is legally required to give you break time to breastfeed, but this break time may not be paid in all workplaces.	159 (52.0)	10 (3.3)	137 (44.8)	99 (32.4)	9 (2.9)	198 (64.7)	25.11	< .001	.20
Your employer is legally required to make a reasonable effort to provide a clean, secure, and private area to breastfeed apart from the bathroom stall.	151 (49.3)	19 (6.2)	136 (44.4)	102 (33.3)	26 (8.5)	178 (58.2)	16.20	< .001	.16
A woman has the legal right to breastfeed in public.	201 (65.7)	7 (2.3)	98 (32.0)	171 (55.9)	11 (3.6)	124 (40.5)	6.35	.04	.10
Before taking this survey, I was aware there are laws related to breastfeeding in the State of AR/AL.	115 (37.6)	154 (50.3)	36 (11.8)	84 (27.5)	160 (52.3)	62 (20.3)	11.84	.003	.14
Federal Law									
The federal law “Break time for nursing mother’s provision” states break time to express breast milk be provided to an employee, up to 1 year after the child’s birth.	91 (29.7)	2 (0.7)	213 (69.6)	67 (21.9)	3 (1.0)	236 (77.1)	5.02	.08	.09
Personal Attitude									
A woman has the ethical right to breastfeed in public.	284 (92.8)	7 (2.3)	14 (4.6)	274 (89.5)	8 (2.6)	23 (7.5)	2.44	.30	.06
Breastfeeding laws matter to me.	259 (84.6)	23 (7.5)	20 (6.5)	224 (73.2)	25 (8.2)	57 (18.6)	20.37	< .001	.18

Note

* indicates correct answer

**Table 11:** Chi-square test results for benefit choice and economic benefits between UAR and AU students.

	UAR (*n* = 306)	AU (*n* = 306)	χ^2^	*p*	Cramer’s *V*
	*n* (%)	*n* (%)			
When feeding an infant less than 6 months, the most beneficial choice is:			*df* = 4 27.81	< .001	.21
Partial breastfeeding	13 (4.2)	9 (2.9)			
Predominant or mostly breastfeeding	130 (42.5)	113 (36.9)			
*Exclusive or only breastfeeding	108 (35.3)	73 (23.9)			
No breastfeeding	1 (0.3)	1 (0.3)			
I don’t know	54 (17.6)	110 (35.9)			
Which of the following is true regarding breastfeeding and economic benefits?			*df* = 2 15.72	< .001	.16
*It has been estimated that the U.S. economy could save over $10 billion per year in pediatric health cost, if 90% of women breastfed for the first year of life.	125 (40.8)	83 (27.1)			
There is little evidence that healthcare costs would change if the majority of mothers breastfed in the U.S.	22 (7.2)	16 (5.2)			
I don’t know.	159 (52.0)	207 (67.6)			

Note

* indicates correct answer

## Discussion

### Knowledge of resources, laws, and current breastfeeding attitudes

Parents stop breastfeeding for a multitude of reasons, including lack of support at home, community, and work [11]. Findings from this study indicate support for breastfeeding initiatives within both the UAR and AU university communities. However, the findings suggest that there is a need for additional education with both students and employees concerning breastfeeding resources and laws. Specifically, there is a knowledge deficit regarding national and state laws protecting the right to breastfeed in public, such as the FLSA, PPACA, and applicable state laws. With the passage of the PUMP Act, it is possible awareness of breastfeeding protection could be accelerated. This was consistent among all participants and when comparing both universities. Similarly, fewer than half of the participants correctly answered questions about breastfeeding rights, the most beneficial feeding choice, and the economic benefits of breastfeeding.

Campus resources, including designated lactation space, exist at both study sites. However, participants, particularly students, lacked knowledge of these resources. Lack of knowledge of breastfeeding and breastfeeding policy, paired with a significant lack of breastfeeding resources, creates an overall deficit in campus support for breastfeeding among the campus community. Despite both study sites offering breastfeeding information, private lactation spaces in a variety of campus buildings, and additional resources, such as lactation spaces at athletic events, awareness of these resources was inadequate. In determining the cause of this lack of awareness, consideration must be given to a lack of signage or publicizing of breastfeeding rooms. Furthermore, not all buildings offer an easily accessible location. A public space to support community breastfeeding should not be the best-kept secret on campus, nor should it be information provided only on a need-to-know basis.

Overall, participants had a positive attitude towards breastfeeding. Overwhelmingly, participants indicated support for breastfeeding rights in public spaces, with about 90% of participants agreeing that there is an ethical right to breastfeed in public. Even with limited knowledge of the law, participants indicated that this right extends to public spaces on college campuses. Providing education for university students and staff is key to creating a better understanding of current laws and regulations concerning breastfeeding in public.

While there were positive attitudes towards public breastfeeding reported at both campuses, the survey response rate was significantly higher at AU. The high participation at AU could indicate a population interested in community breastfeeding education and additional resources. Both universities likely had high participation from nursing students since social media was utilized from student nursing associations who posted the survey link. This does not explain the difference in UAR participants having higher knowledge scores, as the Bachelor of Science in nursing curriculum does not differ significantly. Lastly, the survey was primarily taken by undergraduate students who identified as white and female. The larger number of male participants at AU did not impact the findings.

As expected, employees reported having more children and were older than the student population. Employees with children likely have additional exposure to community resources and breastfeeding. This additional exposure may have supported an increase in knowledge and positive attitudes towards community breastfeeding. UAR reported a slightly higher percentage of participants having children compared to AU, perhaps influencing higher knowledge scores and more positive attitudes regarding public breastfeeding. Students indicated less concern for and knowledge of economics, possibly due to lack of experience with costs associated with caring for children.

Concerning knowledge of breastfeeding resources and attitudes within campus communities, continued inquiry is needed to empower breastfeeding initiatives and create an environment supportive of continued breastfeeding. Advocacy for breastfeeding requires understanding the specific needs of a community and providing the education and resources required to support and encourage an inclusive community. Changing breastfeeding attitudes and perceptions towards normalization requires dedication by breastfeeding advocates and community cooperation. Continued advocacy for and promotion of easily accessible lactation spaces on campus, along with knowledge of applicable breastfeeding laws, is needed and vital to ensure the normalization of breastfeeding in public, which, in turn, will positively impact breastfeeding outcomes. This study serves as an indication of current breastfeeding knowledge and attitudes among those on university campuses in the United States’ southeast/south central regions and can guide breastfeeding education and implementation of future initiatives.

### Limitations

Limitations potentially affecting study outcomes include a homogeneous participant group and lack of control over the sample composition. The target populations were students and employees, but the eligibility criteria were diminished by an unintended bias. The title of the study indicating the topic was breastfeeding could have led potential male participates to assume the study was for females only. This may be due to the nature of the study being inherently more appealing to women than men or those having a stronger opinion on the subject. This limitation poses a high likelihood for selection bias and inadvertently could have greatly decreased male participation. This bias could reduce the validity of the results, targeted at the college campus population. Another limitation of the study is the cross-sectional design: the data reflects one point in time at the selected study locations.

Social desirability bias may also be present when assessing attitudes towards the subject matter. Both students and employees could have reacted to the desire to be positive and support breastfeeding. There were no delimitations; therefore, knowledge or experience with breastfeeding was not considered in the results. The majority of participants were white females, reflecting underrepresentation of diverse populations, making generalizability of the results unreliable for campus populations that differ significantly in ethnicity or diversity. The findings from this study, conducted in the southern United States, may not be generalizable to other university campuses, especially those of different size and geographical location. The survey was also only offered in English, limiting responses from non-English speaking participants.

Although the study began during the COVID-19 pandemic, the survey link was still live during a semester when students were receiving some face-to-face instruction. The timing of the survey is a limitation, however, and warrants further study. Disruption in campus attendance is especially concerning regarding awareness of campus resources, despite information being available online at both universities.

Future studies should include university populations with less homogeneous demographic composition and in various international locations. Additionally, delineation between pumping/expressing milk versus direct breastfeeding in public should be considered.

## Conclusion

While there is general awareness of the benefits of breastfeeding, the recommendation to exclusively breastfeeding for 6 months is not universally known among the college population. There is also a knowledge deficit regarding national and state laws protecting the right to breastfeed in public and the beneficial impact of breastfeeding, including its economic benefit. Although there appears to be a positive attitude toward breastfeeding in public, closer analysis revealed a need for normalization and awareness of legal rights of breastfeeding in public, which aligns with recent studies [4, 5, 8]. Normalization of breastfeeding includes both positive attitudes regarding ethical and legal rights. Although the majority of participants responded positively to the ethical right to breastfeed in public, only about 60% responded positively to the legal right. Campus employees have greater knowledge of the economic cost of not breastfeeding and knowledge of breastfeeding benefits, possibly related to more exposure to breastfeeding personally or in the community. Results of this study support the need for initiatives to promote awareness of the benefits of breastfeeding and continued efforts to eliminate lack of support of breastfeeding in public spaces. Changes to campus policies and support from campus decision makers could promote an environment of inclusion and encourage the university community to utilize campus breastfeeding resources. Including a written policy for lactating students in the student handbook would be an important beginning for most universities, as few have an official policy [3]. Lactation space should be easily accessible, ideally within 10 minutes, and be Americans with Disabilities Act compliant. Campus health centers and community agencies serving those of childbearing age can conduct activities and trainings relating to breastfeeding, along with providing state lactation laws in the workplace. Introductory classes, such as University 101 or Health Science classes should include information on campus lactation policies. Social media campaigns for lactation education must involve the entire campus community. The University of Northern Colorado has developed a toolkit for establishing lactation support on University and College campuses, including funding sources [35]. Utilization of this toolkit or other resources could serve in developing campus breastfeeding initiatives. Supporting the breastfeeding family contributes to better workforce production, increases student and employee retention rates, and raises campus morale. Inclusive campus environments are increasingly important and improving breastfeeding policy and support should involve key stakeholders, especially the lactating family.

## Supporting information

S1 Appendix Breastfeeding in public survey UAR. (DOCX)

S2 Appendix Breastfeeding in public survey AU. (DOCX)

S1 Dataset (DOCX)
