# Supplementary material for: University campus breastfeeding, knowledge, and perceptions of support: An exploratory study
Source: PLoS One. 2023 May 26;18(5):e0285008. doi: 10.1371/journal.pone.0285008 (PMC10218753; doi:10.1371/journal.pone.0285008)
Supplement: S1 Appendix — (DOCX) [file pone.0285008.s001.docx]

Appendix 1

Breastfeeding in Public: Knowledge and Perceptions on a University Campus: UA

Breastfeeding Survey (Arkansas)

Start of Block: Default Question Block

Q1 Please rate the extent to which you agree or disagree with the following statements.

|  | Strongly Agree | Agree | Neither agree nor disagree | Disagree | Strongly Disagree |
| --- | --- | --- | --- | --- | --- |
| I would be comfortable if a mother were to breastfeed in public, such as at work or in a restaurant. |  |  |  |  |  |
| Mothers should take measures to cover themselves when breastfeeding in public. |  |  |  |  |  |
| Mothers should avoid breastfeeding in public. |  |  |  |  |  |
| In my culture, breastfeeding in public is considered normal. |  |  |  |  |  |
| I would feel embarrassed to see a mother breastfeed in public. |  |  |  |  |  |

Q2 Please rate the extent to which you agree or disagree with the following statements.

|  | Strongly agree | Agree | Neither agree nor disagree | Disagree | Strongly disagree |
| --- | --- | --- | --- | --- | --- |
| The benefits of breastfeeding last only as long as the baby is breastfed. |  |  |  |  |  |
| Breastfeeding increases mother-infant bonding. |  |  |  |  |  |
| Formula feeding is more convenient than breastfeeding. |  |  |  |  |  |
| Breast milk is lacking in iron. |  |  |  |  |  |
| Formula fed babies are more likely to be overfed than breastfed babies. |  |  |  |  |  |
| Breast milk is cheaper than formula. |  |  |  |  |  |
| Formula feeding is the better choice if the mother plans to go back to work. |  |  |  |  |  |
| Mothers who formula feed miss one of the great joys of motherhood. |  |  |  |  |  |
| Breastfed babies are healthier than formula fed babies. |  |  |  |  |  |
| Breastfed babies are more likely to be overfed than formula fed babies. |  |  |  |  |  |
| Fathers feel left out if a mother breastfeeds. |  |  |  |  |  |
| Breast milk is the ideal food for babies. |  |  |  |  |  |
| Breast milk is more easily digested than formula. |  |  |  |  |  |
| Formula is as healthy for an infant as breast milk. |  |  |  |  |  |
| Breastfeeding is more convenient than formula. |  |  |  |  |  |
| Breast milk is cheaper than formula. |  |  |  |  |  |
| A mother who occasionally drinks alcohol should not breastfeed her baby. |  |  |  |  |  |

Q3 Please respond to the following statements based on your knowledge of your college campus.

|  | True | False | I don't know |
| --- | --- | --- | --- |
| Designated breastfeeding areas exist across campus. |  |  |  |
| There are at least 13 designated breastfeeding areas on campus. |  |  |  |
| Following the State law, designated space, including employee office space, must be available on campus. |  |  |  |
| A campus map of breastfeeding areas is available online. |  |  |  |
| Private breastfeeding space at some university athletic events is currently offered. |  |  |  |

Q4 Please respond to the following statements based on your knowledge of laws in the state of Arkansas.

|  | True | False | I don't know |
| --- | --- | --- | --- |
| Your employer is legally required to give you break time to breastfeed, but this break time may not be paid in all workplaces. |  |  |  |
| Your employer is legally required to make a reasonable effort to provide a clean, secure, and private area to breastfeed apart from the bathroom stall. |  |  |  |
| A woman has the legal right to breastfeed in public. |  |  |  |
| Before taking this survey, I was aware there are laws related to breastfeeding in the state of Arkansas. |  |  |  |

Q5 Please respond to the following statements based on your personal attitude.

|  | Agree | Disagree | I don't know |
| --- | --- | --- | --- |
| A woman has the ethical right to breastfeed in public. |  |  |  |
| Breastfeeding laws matter to me. |  |  |  |

Q6 True or false: The Federal law “Break Time for Nursing Mother’s Provision” states break time to express breast milk be provided to an employee, up to 1 year after the child’s birth.

- True
- False
- I don't know

Q7 When feeding an infant less than 6 months, the most beneficial choice is

- Partial breastfeeding
- Predominant or mostly breastfeeding
- Exclusive or only breastfeeding
- No breastfeeding
- I don't know

Q8 Which of the following is an adequate duration of breastfeeding?

- Until the baby is 6 months old
- Until the baby is 12 months old
- Until the baby is 18 months old and beyond
- I don't know

Q9 Which of the following is true regarding breastfeeding and economic benefits?

- It has been estimated that the U.S. economy could save over $10 billion per year in pediatric health cost, if 90% of women breastfed for the first year of life.
- There is little evidence that healthcare costs would change if the majority of mothers breastfed in the U.S.
- I don't know

Q10 Please select your status at the university

- Undergraduate Student
- Graduate Student
- Faculty
- Administrative Support/Staff
- Other university employee

Q11 Please select your gender

- Male
- Female
- Gender diverse
- Prefer not to answer

Q12 What is your ethnicity?

- White
- Hispanic or Latino
- Black or African American
- Native American or American Indian
- Asian or Pacific Islander
- Other

Q13 Do you have children?

- Yes
- No

Q14 How old are you (in years)?

________________________________________________________________

Q 15: If you wish to be entered in the drawing for one of ten, $50 Amazon gift cards, please enter your email address here :
