# Supplementary material for: University campus breastfeeding, knowledge, and perceptions of support: An exploratory study
Source: PLoS One. 2023 May 26;18(5):e0285008. doi: 10.1371/journal.pone.0285008 (PMC10218753; doi:10.1371/journal.pone.0285008)
Supplement: S1 Dataset — (DOCX) [file pone.0285008.s003.docx]

DATA SET

Upon acceptance of publication, the data set can be retrieved from Figshare via the following:

**doi #: 10.6084/m9.figshare.20406168.**
